# Examining accident reports involving autonomous vehicles in California

**DOI:** 10.1371/journal.pone.0184952

**Published:** 2017-09-20

**Authors:** Francesca M. Favarò, Nazanin Nader, Sky O. Eurich, Michelle Tripp, Naresh Varadaraju

**Affiliations:** 1 Department of Aviation and Technology, San Jose State University, San Jose, California, United States of America; 2 RiSA^2^S Research Center, San Jose State University, San Jose, California, United States of America; Chongqing University, CHINA

## Abstract

Autonomous Vehicle technology is quickly expanding its market and has found in Silicon Valley, California, a strong foothold for preliminary testing on public roads. In an effort to promote safety and transparency to consumers, the California Department of Motor Vehicles has mandated that reports of accidents involving autonomous vehicles be drafted and made available to the public. The present work shows an in-depth analysis of the accident reports filed by different manufacturers that are testing autonomous vehicles in California (testing data from September 2014 to March 2017). The data provides important information on autonomous vehicles accidents’ dynamics, related to the most frequent types of collisions and impacts, accident frequencies, and other contributing factors. The study also explores important implications related to future testing and validation of semi-autonomous vehicles, tracing the investigation back to current literature as well as to the current regulatory panorama.

## 1. Introduction

Autonomous Vehicle (AV) technology is quickly expanding its market, fostered by the potential and promise of addressing important transportation issues, such as: (i) the improvement of roads safety, where human error is estimated to account for 94% of the total accidents [1]; (ii) the improvement of the commute experience, allowing to re-allocate part of the commute time to tasks other than driving, and with the potential to shorten the commute once the car takes care of parking for itself [2]; (iii) the long-sought improvement of mobility for everyone, enabling differently abled people to access transportation and improving independence [3]; (iv) the potential for fuel savings and more manageable parking arrangements, which among other things help classify this type of technology as a “green” and eco-friendly alternative to more traditional means of transportation [2].

Together with the thrill associated with advancement in technology also comes the struggle to make these systems safe, and the effort for certifying them to ensure the safety of the consumer and the public. The AV database of the National Conference of State Legislation of the United States currently displays 35 State Senate Bills as “pending” with regards to autonomous vehicles testing and operations on public roads, [4]. At the heart of the safety concerns is the role of the human driver in his/her interface with a car that can be subject to brittle automation.

Roughly speaking, an autonomous vehicle is any vehicle that adopts a technology capable of *supporting and assisting a human driver* in the tasks of: 1) controlling a vehicle (and its main functions of steering and controlling its speed); and 2) monitoring the surrounding environment (e.g., other vehicles/pedestrians, traffic signals, road markings, etc.). The two functions are clearly interconnected and depend on each other, given that the execution of particular control functions (e.g., accelerating or decelerating) will depend on inputs and signals received from the surrounding environment (e.g., a traffic light turning red).

**Fig 1:**
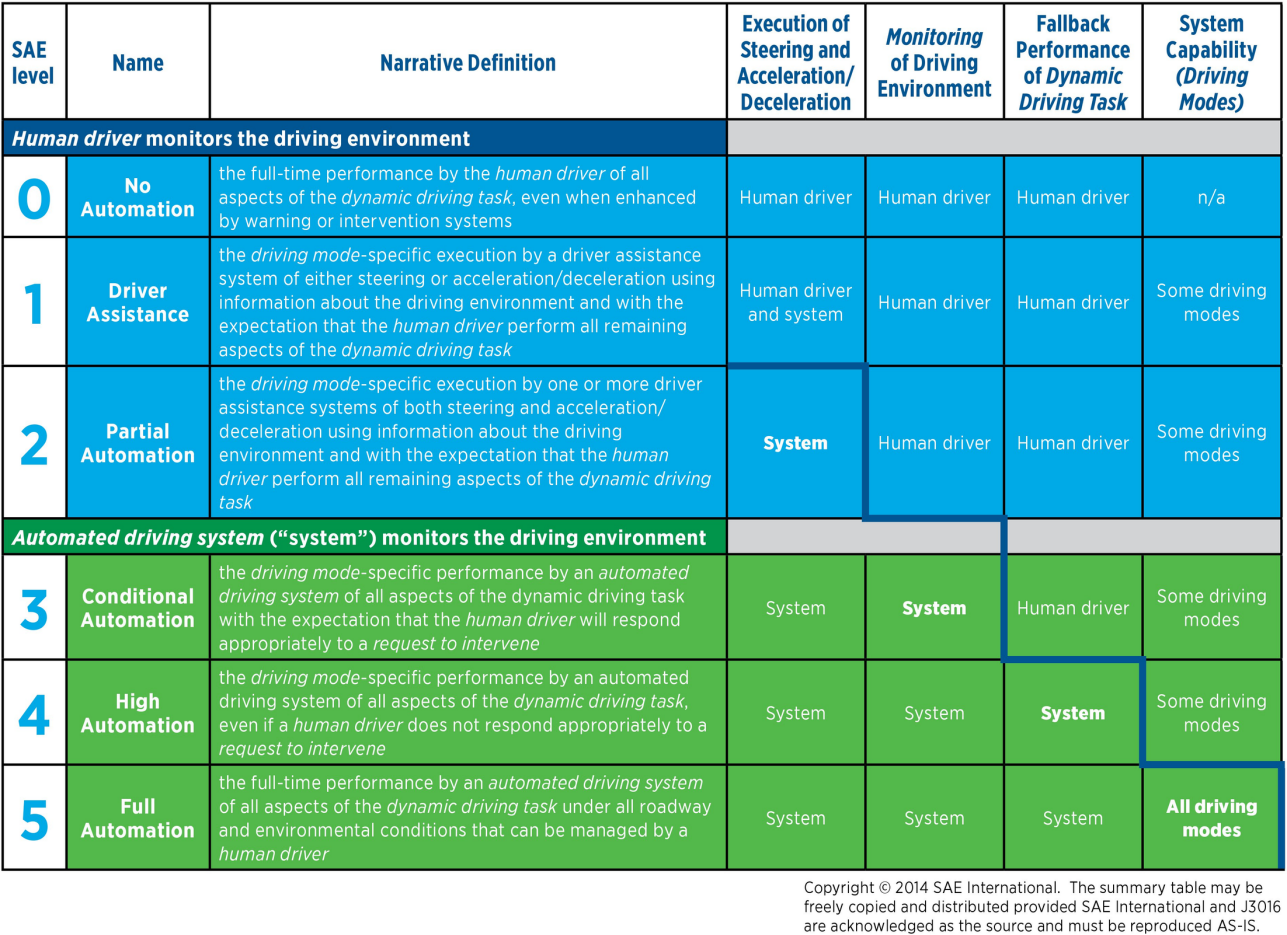
AV levels of automation. Reproduced AS-IS with permission from SAE-International J3016^TM^, [5].

A more precise answer to the fundamental question of “what is an AV” comes from the Society of Automotive Engineers (SAE). SAE defines 6 levels of autonomy which revolve around the extent to which the Autonomous Technology (AT) is capable of supporting and assisting the driving tasks [5]. Fig 1 provides a summary of the definition of the six levels of autonomy, nowadays commonly accepted by car manufacturers as well as regulators and policy-makers.

The levels of autonomy go from 0 (no automation) to 5 (full unrestricted automation). SAE identifies four factors that affect the applicability of the automation levels:

1. The agent responsible for executing steering and throttle control (human driver vs. AT);
2. The agent responsible for monitoring the external environment;
3. The agent responsible to serve as “back-up” when a failure prompts a disengagement of the AT;
4. The driving modes in which autonomous operations are allowed (i.e., all vs. restricted to particular conditions–e.g., nice weather).

The third factor is responsible for the creation of two big categories of AV systems, that are currently at the center of public debates: semi-autonomous vehicles vs. fully-autonomous vehicles. Levels 1 through 3 are regarded as “semi-autonomous” due to the fallback performance (or back-up) of the driving tasks placed on the human driver. Conversely, Levels 4 and 5 are fully automated. Specifically, Levels 4 and 5 can be regarded as “restricted full autonomy” and “unrestricted full autonomy” respectively, with restrictions being placed on the system capabilities in different driving modes and external conditions (e.g. fully-autonomous vehicles that can only operate in daytime or under clear weather conditions).

Both semi- and fully-autonomous cars can be subject to disengagement modes. In semi-autonomous vehicles a human pilot is allowed to cooperate with the software that acts as the “brain” of the vehicle whenever he/she wishes to do so. In fully-autonomous design options, full-authority on the system movement is instead handled by the software at all times. During disengagement of the autonomous technology (AT) “brain”, the car control authority shifts from autonomous to manual mode, thus handing the control back from the software to the human driver. In the safety-critical situations of a disengagement, it is important to ensure that the human driver has enough time to react and respond effectively to the request to control the vehicle.

In an effort to promote safety, the California Department of Motor Vehicles (CA DMV) has mandated that trained human drivers be behind the wheel at all times during testing on public roads, regardless of the level of autonomy of the vehicle. This implies that fully-autonomous vehicles are currently retrofitted to allow for a steering wheel, control pedals, and a human driver in the AV. Furthermore, to promote transparency to consumers, the DMV had mandated that two types of report be drafted and made available to the public following failures of the autonomous technology during testing [6]. The first type of reports is a concise list of all occurrences of AT disengagements, meaning a summary of failure events in which either autonomously or manually (i.e., initiated by the human driver) the autonomous “brain” of the car disengages and the control reverts back to the human driver. The second type of reports provides a more detailed summary of events for those occurrences in which a collision and/or damage to property and injuries occur.

**Fig 2:**
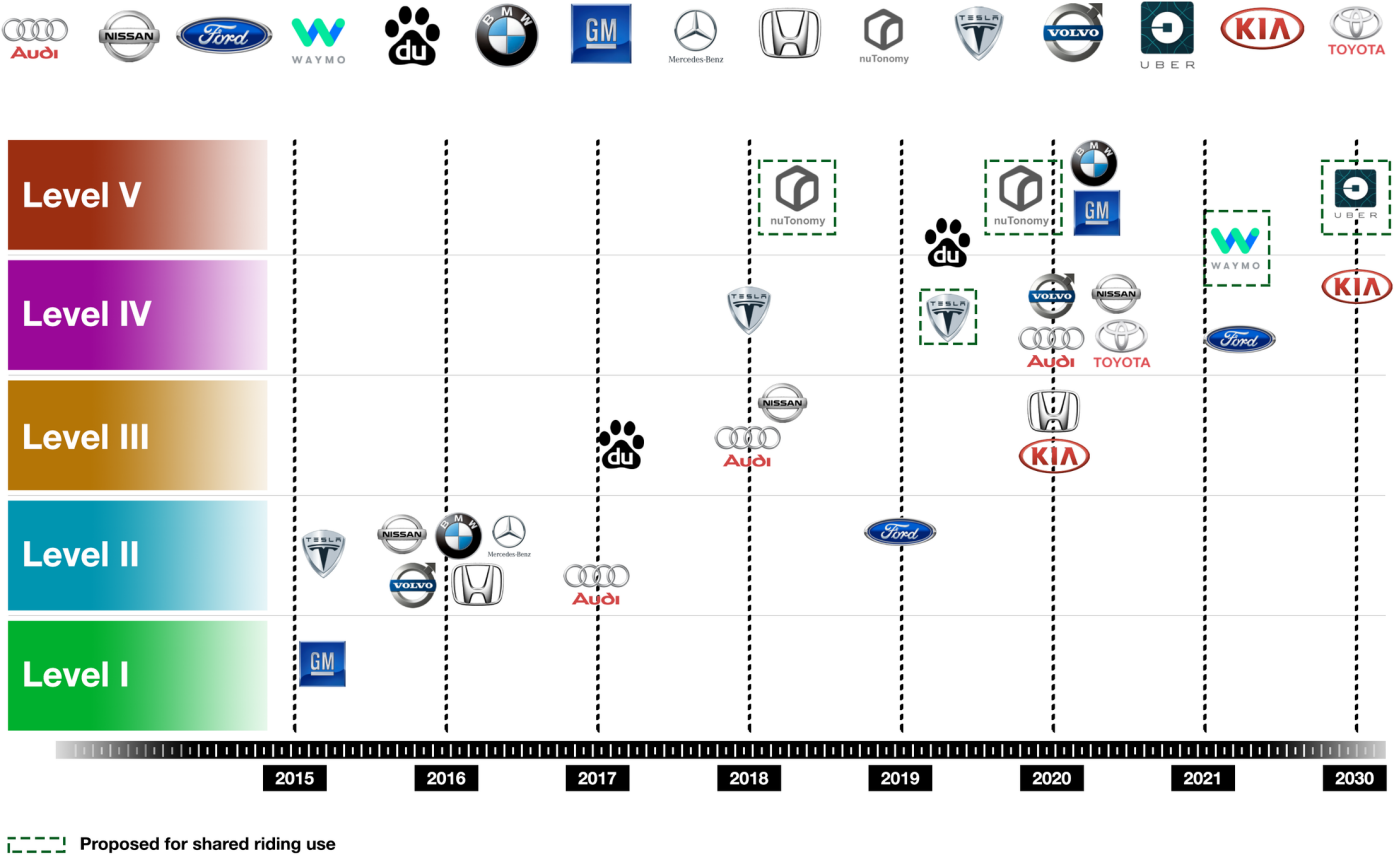
Overview of AV market, 2015–2030 estimated timeline. Not meant to be exhaustive. The data points were estimated based on media articles from wired.com, motortrend.com, forbes.com, bbc.com, and from manufacturers’ websites and public statements.

As of May 2017 there are thirty manufacturers that acquired permission from the CA DMV to begin testing of AV on CA public roads. Manufacturers are targeting different levels of autonomy, with semi-autonomous vehicles currently in the lead. Fig 2 provides an overview of how the AV market of is shaping, with estimated timelines and levels of automation targeted by several major manufacturers.

The original draft of the CA DMV regulations for deployment prohibited manufacturers from selling fully-autonomous vehicles [6], allowing deployment of only semi-autonomous vehicles with a back-up driver. The regulation draft highlights the role of the human driver, who is responsible *“for monitoring the safe operation of the vehicle at all times*, *and must be capable of taking over immediate control in the event of an autonomous technology failure or other emergency”* [7].

The recent crash of a Tesla Model S in May 2016 [8], has heightened the debate of whether more stringent regulations might be needed, tightening the certification requirements for semi-autonomous vehicles. Many automakers have advanced the hypothesis that skipping Level 3 altogether and aiming directly for Level 5 (although on a longer timeline) might be a safer option [9], which would also allow regulators to pick up the pace with the AV technology.

Stricter regulations that prevent deployment at the present time are one possible solution to address the issue. At the same time though, a careful study of the available data allows gathering insight into the ways these systems are failing, and possibly better inform future regulations. The goal of this study is thus to analyse the data related to AV accidents provided to the CA DMV by different manufacturers that are testing autonomous vehicles in California (testing data from 2014 to 2017). The in-depth focus of this paper is on the detailed AV accident reports. The data provides important information on AV accidents dynamics, related to most frequent types of collisions and impacts, accident frequencies, and other contributing factors. The results obtained are traced back, whenever possible, to gaps and limitations within the current literature and the regulatory panorama.

The remainder of this paper is structured in the following way. Section 2 provides an overview of the CA DMV database, as well as a current literature review on the topic. Section 3 examines in detail the full accident reports of collisions among AVs testing on public California roads. Section 4 concludes this work.

## 2. The database and previous studies

Whether forced by design choices or due to insufficient information regarding the context of a particular situation, an autonomous car can suffer from what it is called a “disengagement mode”. During disengagement, the full control and authority of the car movement is handed from the autonomous software to the human driver.

The CA DMV currently mandates that reports for such disengagements during testing and/or field operations be drafted and made available to the public. It is important to understand that a disengagement does not necessarily lead to an accident. The DMV has thus created two separate databases, depending on the type of outcome of the particular occurrence:

1. **Autonomous Vehicle Disengagement Reports Database [10]**: this database includes data related to all disengagement reports that occurred during testing on CA public roads between September 2014 and January 2017 as reported by Bosch, Delphi Automotive, Google, Nissan, Mercedes-Benz, Tesla Motors, BMW, GM, Ford, Honda, and Volkswagen Group of America. This database lends itself to statistical analysis, and currently includes a total of 5,326 data points. In most instances, the AT disengagement does not lead to an actual accident. Manufacturers that are testing on CA public roads are mandated to update their disengagement list each year. This database includes both accident occurrences in which an AT disengagement occurred (as a simple data point), and situations in which the off-nominal condition of the disengagement did not lead to any serious consequence (the vast majority of them, considering that only 26 accidents have been reported so far). Each manufacturer provides data on the mileage driven each month, along with specific details related to each disengagement (e.g., weather conditions, brief description of the cause of disengagement, road type, and other relevant information depending on the case).
2. **Report of Traffic Accidents Involving Autonomous Vehicles Database [11]**: this database provides more descriptive and detailed reports for actual accidents (i.e., minor and/or major collisions with damage to public property and/or serious injuries to people) that occurred in the 2014–2017 timespan during testing of autonomous cars on CA public roads. Manufacturers include Google, General Motors, Cruise Automation, Delphi, and Nissan. The database at time of publication of this work consists of 26 events. Due to their limited number, these occurrences can be analyzed in a deeper and more detail context. This analysis will constitute the core of Section 3 and of the present paper.

The disengagement database has been the subject of study of a number of media articles and tech blogs. Data up to November 2015 was preliminarily analyzed and published in [12]. The research published in [12] brought forward four main conclusions, which can be summarized as follows:

1. The number of accidents observed had a significant high correlation with the autonomous miles traveled (i.e., the more cumulative miles traveled, the more cumulative accidents);
2. Of the two companies (Google and Mercedes-Benz) analyzed in [12] for a study on reaction times, an average reaction time of 0.83 seconds was obtained;
3. Lack of trust was found to increase the likelihood to take control of the vehicle;
4. The reaction times were found to increase with increased vehicle miles travelled, suggesting an increased level of trust with increased mileage.

The work presented in this paper builds up and expands on what was concluded in [12] and revisits some of the conclusions of a previous conference presentation by the authors [13]. With regards to [12]: i) an in-depth analysis of traffic accidents involving autonomous vehicles is featured in this paper, whereas [12] treated the disengagements database in detail with only a brief overview of the accidents; (ii) a bigger database is here employed, with data up to March 2017 (i.e., at the time of publication of [12], only 2,891 disengagements and 12 accidents had occurred, vs. the current database of 5,326 disengagements and 26 accident reports). Moreover, the results contained in [12] will be used to validate some of our conclusions, and repetitions will be avoided unless necessary for clarity of exposition. To the authors’ knowledge, there are no other technical publications that feature the analysis of the CA DMV AVs databases at this time.

## 3. Analysis of accident reports

### 3.1 Reporter’s overview

**Fig 3:**
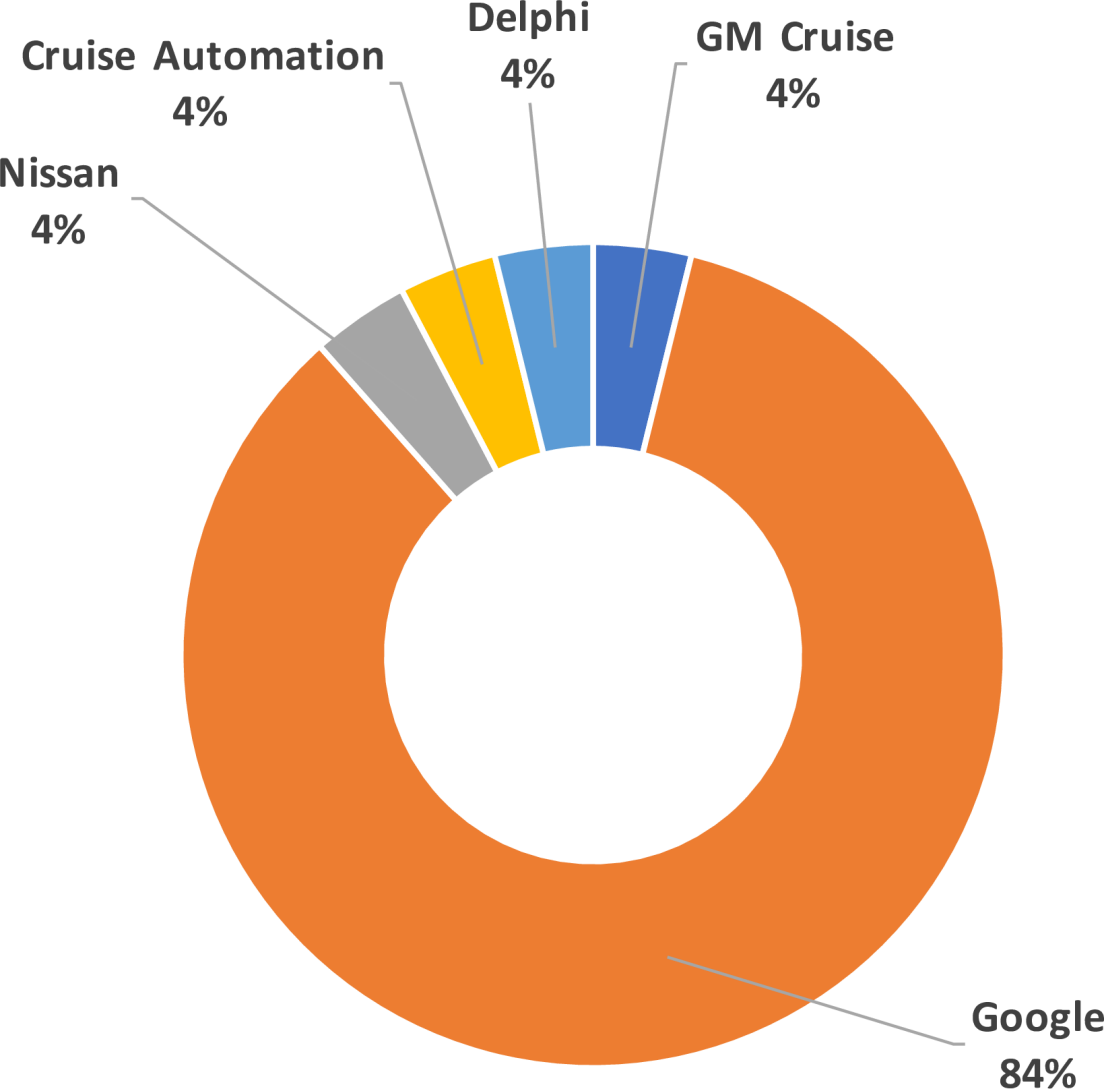
Breakdown of AV accident reporters. Data from September 2014 to March 2017.

As of May 2017, the CA DMV has issued 30 permits to AVs manufacturers for testing on CA public roads. Of those, only 5 have reported traffic accidents, as mandated by [6] for occurrences that lead to “[…] vehicles in any manner involved in an accident originating from the operation of the autonomous vehicle on a public road that resulted in the damage of property or in bodily injury or death”. Traffic accidents have to be reported within 10 business days from the occurrence [6]. The five manufacturers that have reported traffic accidents are listed in Fig 3, along with relative frequency of the reports per manufacturer.

**Fig 4:**
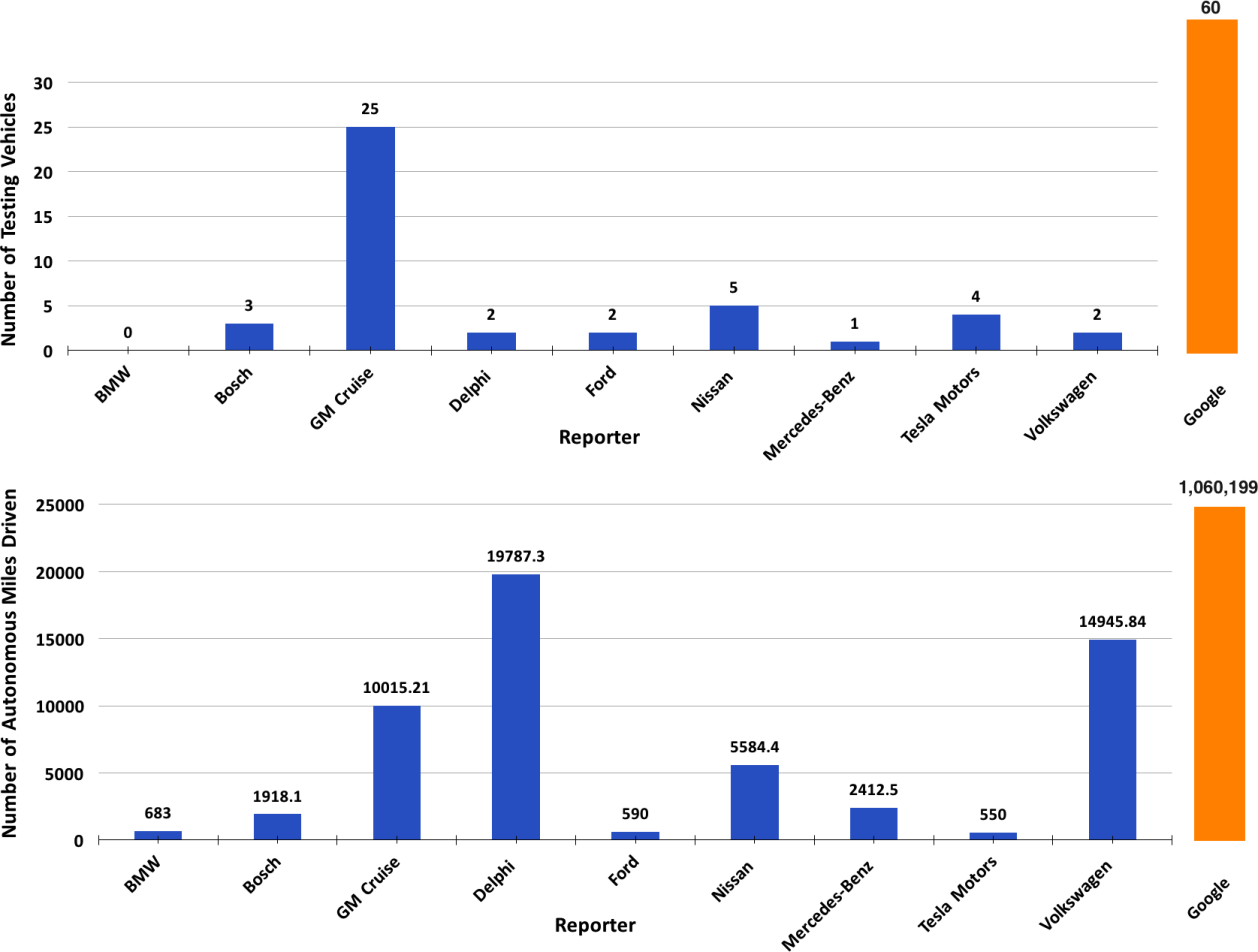
Number of vehicles and total miles travelled for each reporting agency. BMW omits fleet size, here reported as a zero.

As gathered from Fig 3, Google’s reports of AV accidents account for 84% of the total. This disparity is due to the much larger effort (when compared to the other reporters) in terms of fleet size and mileage travelled. Fig 4 provides an overview of mileage travelled and number of vehicles employed by manufacturers (with Google data presented out of scale for easier interpretation of the data from the other agencies). Google (now Waymo) fleet is composed of 60 vehicles (as of 2017) [10], including both Google’s own prototype of self-driving car and retrofitted vehicles (currently using Lexus RX450s [14]). Google’s testing campaign, in terms of vehicles employed and miles travelled, is considerably larger than the other manufacturers currently under permit.

### 3.2 Traffic accidents analyses: Frequencies, dynamics, and damage analysis

#### 3.2.1 Accidents’ overview

The authors in [12] opted for including a summarizing table for the traffic accident reports. An expanded and more detailed version of a similar feature is provided here for all 26 occurrences in Table 1.

**Table 1:** Summary table of reported AV accidents. Data from September 2014 to March 2017. Time of the day is provided in 24-hours format. AM, PM indicate before noon and after noon time, when exact time of day is not available. “V” stands for vehicle and is followed by the number of the vehicle involved (e.g., V#2, second vehicle other than the AV). Status of vehicles and relative direction formatting is as follows: “AV status / V#2 status; relative direction”. Relative direction formatting is as follows: “\” if vehicles travelled in the same direction, “|” if perpendicular” (example: Moving/Stopped; |, meaning AV moving, Vehicle #2 stopped; vehicles traveling in perpendicular directions).

ID	Date & Time	Company	Make	Autonomous Mode?	Status of vehicles and relative direction	AV damage	Other vehicle damage	Injuries	Contributing Factor
**1**	2/16/17, 8:38	GM Cruise	Chevy	Yes	Moving/Moving; \	Rear bumper, minimal	V#2: front and rear bumpers, minor. V#3: front end, minor	No	Other vehicles manoeuvres
**2**	12/11/16, 11:36	Google	Lexus	Manually disengaged	Moving/Moving; \	Driver's side door, minor	Front bumper, minor	No	Other vehicle manoeuvres
**3**	10/26/16, 10:27	Google	Google	Yes	Moving/Moving; \	Rear hatch, minor	Front bumper, minor	No	Other vehicle manoeuvers
**4**	09/23/16, 11:58	Google	Lexus	Manually disengaged	Moving/Moving; |	Front and rear doors, substantial	Front end, substantial	No	Other vehicle manoeuvers
**5**	09/14/16, 15:06	Google	Google	Manually disengaged	Moving/Moving; \	Rear tire and door passenger side, minor	Front bumper and driver side fender, moderate	No	Other vehicle manoeuvers
**6**	09/07/16, 18:47	Google	Google	Yes	Moving/Moving; \	Rear bumper and hatch, minor	Front bumper and right headlight, minor	No	Other vehicle manoeuvers
**7**	09/02/16, 10:41	Google	Google	Yes	Stopped/Moving; \	Rear bumper and hatch, moderate	Front bumper, minor	No	Other vehicle manoeuvers
**8**	08/16/16, PM	Google	Google	Yes	Stopped/Moving; \	Rear bumper and hatch, moderate	Front bumper, minor	No	Other vehicle manoeuvers
**9**	08/08/16, PM	Google	Google	No	Stopped/Moving; \	Rear hatch and bumper, minor	None	No	Other vehicle manoeuvers
**10**	07/15/16, 15:26	Google	Google	Yes	Stopped/Moving; \	Rear hatch and sensor, minor	None	No	Other vehicle manoeuvers
**11**	05/10/16, 15:00	Nissan	Nissan	No	Moving/Moving; \	Front scuff marks, minor	Rear bumper scuff marks, minor	No	Operator’s decision-making
**12**	05/04/16, 21:45	Google	Google	No	Moving/ N/A; N/A	Side, minor	N/A	No	Operator’s decision-making
**13**	04/28/16, 17:35	Google	Google	Yes	Stopped/Moving; \	Rear bumper, minor	Front bumper, minor	No	Other vehicle manoeuvers
**14**	04/07/16, AM	Google	Google	Yes	Stopped/Moving; \	None	Left side mirror slightly folded in	No	Other vehicle manoeuvers
**15**	02/14/16, PM	Google	Lexus	Yes	Moving/Moving; \	Left front fender, wheel and driver-side sensors	None	No	AV prediction/bus driver
**16**	01/08/16, 13:41	Cruise Automation	Nissan	Manually disengaged	Moving/Stopped; \	Front left quarter panel area, minor	Front left quarter panel area, minor	No	Operator’s decision-making/ AV prediction
**17**	11/02/15, 14:50	Google	Lexus	Yes	Stopped/Moving; \	Rear bumper, minor	Passenger side headlight, vehicle hood and front bumper, minor	No	Other vehicle manoeuvers
**18**	08/20/15, 9:36	Google	Lexus	Manually disengaged	Moving/Moving; \	Rear left bumper, minor	Front end, moderate, towed	AV driver: back, minor	Other vehicle manoeuvers
**19**	07/01/15, 17:16	Google	Lexus	Yes	Stopped/Moving; \	Rear bumper, minor	Front end, substantial	AV passengers: whiplash; V#2 driver: neck and back, minor	Other vehicle manoeuvers
**20**	06/18/15, 11:15	Google	Lexus	Yes	Stopped/Moving; \	Rear bumper, minor scrapes	Front bumper, minor scrapes	No	Other vehicle manoeuvers
**21**	06/04/15, 8:54	Google	Lexus	Yes	Stopped/Moving; \	None	None	No	Other vehicle manoeuvers
**22**	05/30/15, 12:00	Google	Lexus	Yes	Stopped/Moving; \	Rear sensor and bumper, minor	None	No	Other vehicle manoeuvers
**23**	04/27/15, 16:57	Google	Lexus	Yes	Stopped/Moving; \	None	None	No	Other vehicle manoeuvers
**24**	04/07/15, AM	Google	Lexus	Yes	Moving/Moving; \	Body damage, minimal	None	No	Other vehicle manoeuvers
**25**	02/26/15, Am	Google	Lexus	Manually disengaged	Moving/Moving; |	Right rear quarter panel and right rear wheel	N/A	No	Other vehicle manoeuvers
**26**	10/14/14, 19:27	Delphi	Audi	No	Stopped/Moving; |	Damaged fender and front bumper	N/A	No	Other vehicle manoeuvers

Table 1 is reconstructed based on the information provided in the accident reports. Per regulations [6], AV accident reporters have to provide details of the accident occurrence including:

- Number of vehicles involved
- Status of the vehicle(s) (e.g., moving, stopped)
- Parties involved other than vehicles (e.g., pedestrian, bicycles)
- Injuries and property damage
- Description of the accident’s dynamics, including specifying whether the AV was driving in autonomous or conventional (i.e., manual) mode.

**Fig 5:**
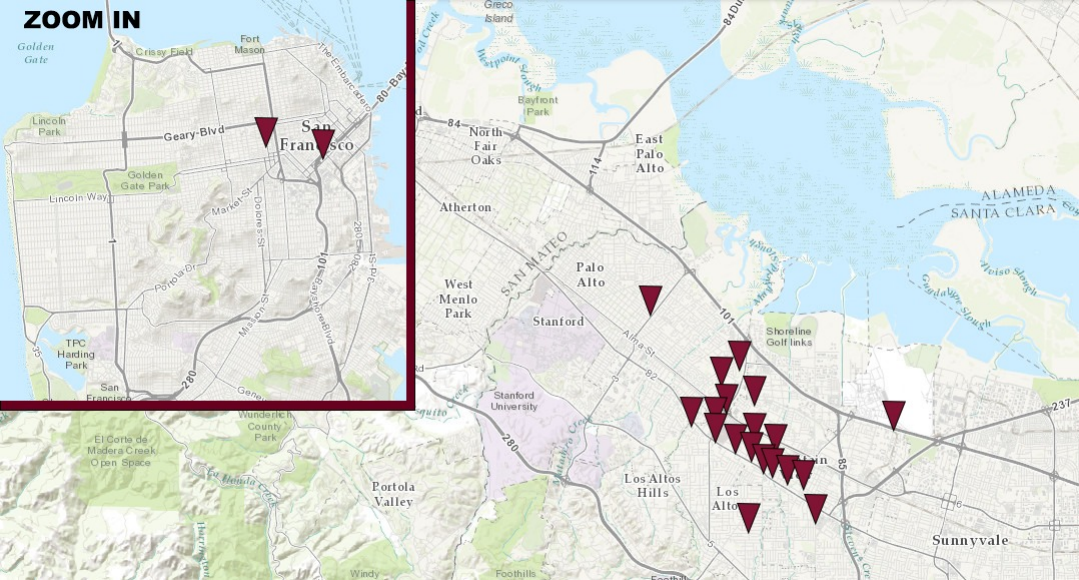
Mapping of AV accidents’ locations.

**Fig 6:**
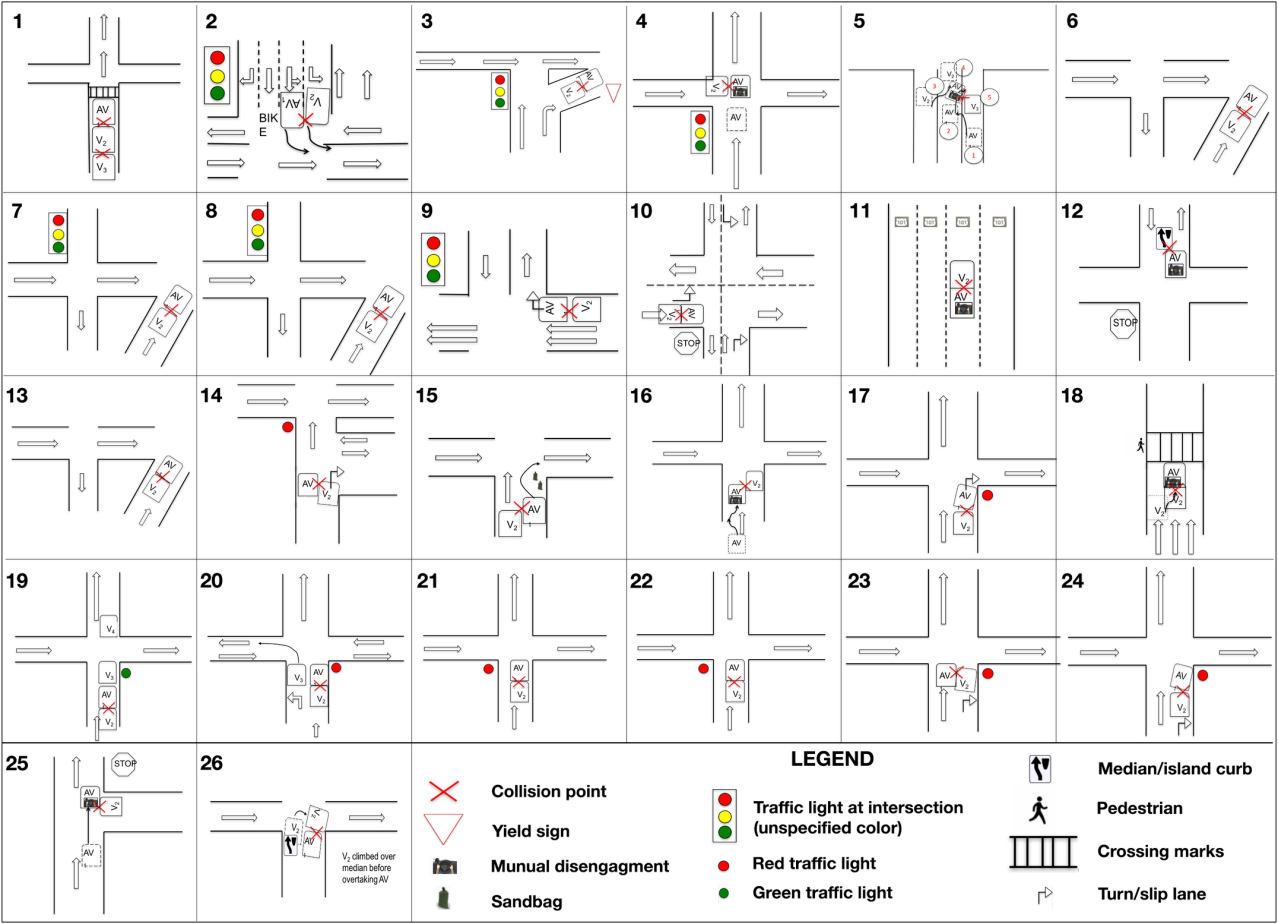
Visual reconstruction of AV accidents’ dynamics.

The accidents’ descriptions include information on the location of the accident. Based on this information it was possible to locate “hot spots” for AV accidents in the San Francisco Bay Area (as shown in Fig 5), and, after inspection of the intersections involved in the accident sequences, reconstruct visually the dynamics of the accidents, with the relative positioning of the vehicles. Such reconstruction is presented in Fig 6, which provides the visual counter-part of Table 1. Fig 6 also attempts at showing the path followed by the vehicles involved in the accidents, highlighting their relative position at two specific instants of time: i) the time at which the AT was disengaged (either manually or due to AT failure); ii) the time of the collision. The first situation is not always represented, as not all vehicles underwent a disengagement during the accident sequence (more details on this point are provided in Section 3.3).

#### 3.2.2 Accidents dynamics

**Fig 7:**
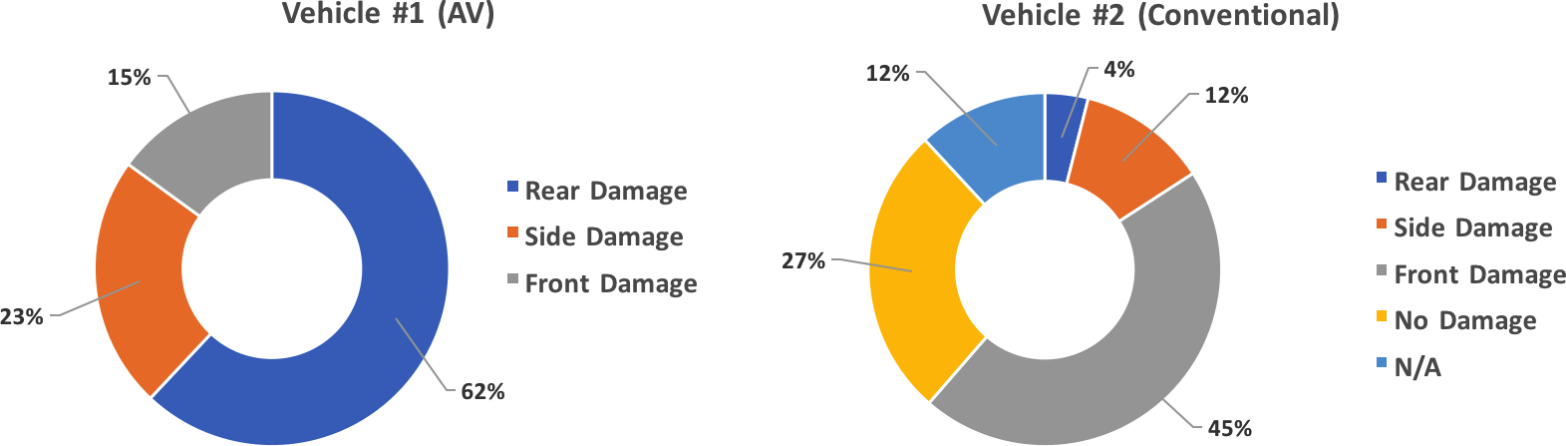
Damage location breakdown for vehicles involved in collisions.

The sketches of Fig 6 point out that most of the accidents are “rear-end” type of collisions, with the AV hit from the rear by an upcoming vehicle. Interestingly, Fig 6 and the descriptions of Table 1 also indicate that in no case the vehicles involved in the collisions were traveling in opposite directions. Fig 7 summarizes the location of the damage for both the AV and the second vehicle involved in the collision.

According to the National Highway Traffic Safety Administration (NHTSA) and the Bureau of Transportation Statistics, 94% of the accidents involving conventional vehicles (i.e., without AT) are related to human errors [1] (with one quarter of those due to distraction, according to [15]). NHTSA estimates that about 30% of conventional motor vehicles accident are rear-end/fender-bender type [16] that involve highly distracted drivers. Careful considerations need to be addressed when comparing the 62% indicated in Fig 7 to the 30% reported by NHTSA for conventional vehicles. At a first-glance it may appear that AV’s probability of rear-end collisions doubles that of conventional vehicles. Our interpretation of this datum however is that the results of Fig 7 suggest that AV technology is capable of preventing all other accident typologies effectively, leaving rear-end collisions with the AV in front the most important failure scenario to be addressed next by manufacturers. Plenty of strategies in fact exist to prevent rear-end collisions when an AV is in the back. Safety margins based on the minimum distance will lead to the deployment of automatic breaking whenever the AV driver gets unintentionally too close to the leading front vehicle, thus limiting the amount of “front damage” scenarios that we see in Fig 7 (note that automatic assisted breaking is an available feature in many Level 2 vehicles currently on the market). Table 1 indicates that in only one occasion the AV was responsible for a rear-end collision, hitting a conventional vehicle from behind (accident number 11 in Fig 6 and Table 1). In this situation however, the AV was driven manually on highway 101, and the probable cause is attributed to flawed operator’s decision-making. Auto-braking is indeed an easily achievable target, making rear-end collisions with an AV in the rear virtually impossible when automation is properly engaged. Current semi-AVs on the market establish ample safety margins on distances that should always be kept between the rear and the front vehicles, automatically activating brakes as soon as those safety margins are no longer respected.

**Fig 8:**
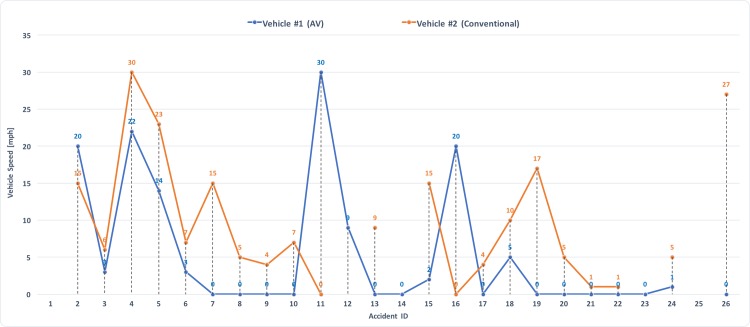
Speed distribution for vehicles involved in the AV accidents. The x-axis shows Accidents Identification following the IDs indicated in Table 1 and Fig 6.

**Fig 9:**
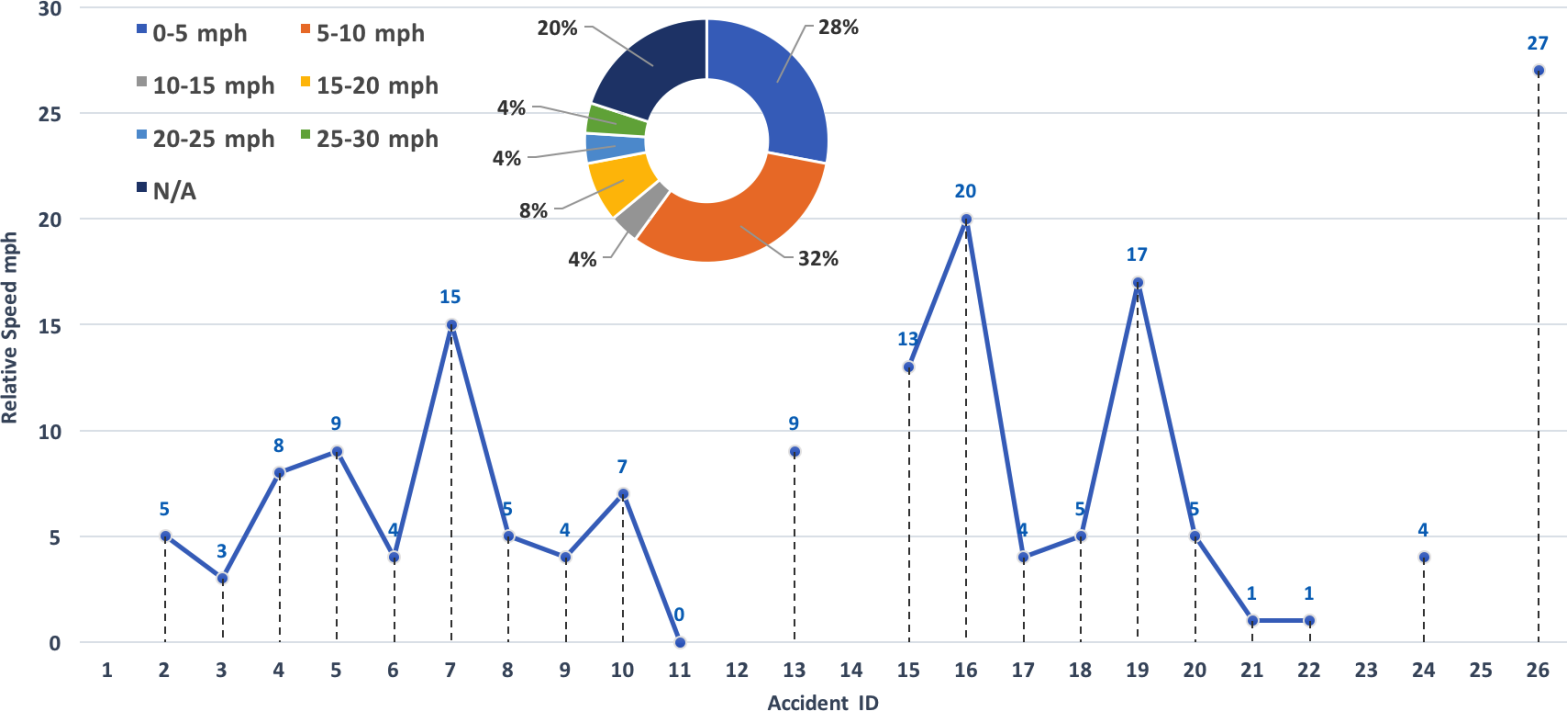
Relative speed of the colliding vehicles in reported AV accidents and breakdown. The x-axis shows Accidents Identification following the IDs indicated in Table 1 and Fig 6.

The data in Figs 6 and 7 lends itself to an analysis of the relative motion of the two vehicles, to better understand the dynamics of the accident. Fig 8 shows the traveling speed of the vehicles involved in the accidents. Not all reports indicated the vehicles speeds, so that gaps are left where the information was not available. As mentioned, the majority of the accidents were rear-end “fender-bender” types, and the speed trends indicates that in most situations the AV was at zero or close-to-zero speed. To gather a sense of the impact force, it is possible to plot the relative speeds between the two vehicles involved in the collision. Doing so leads to Fig 9, where a pie chart also provides a break-down of the relative speeds in six categories (i.e., from low impact to high impact).

**Fig 10:**
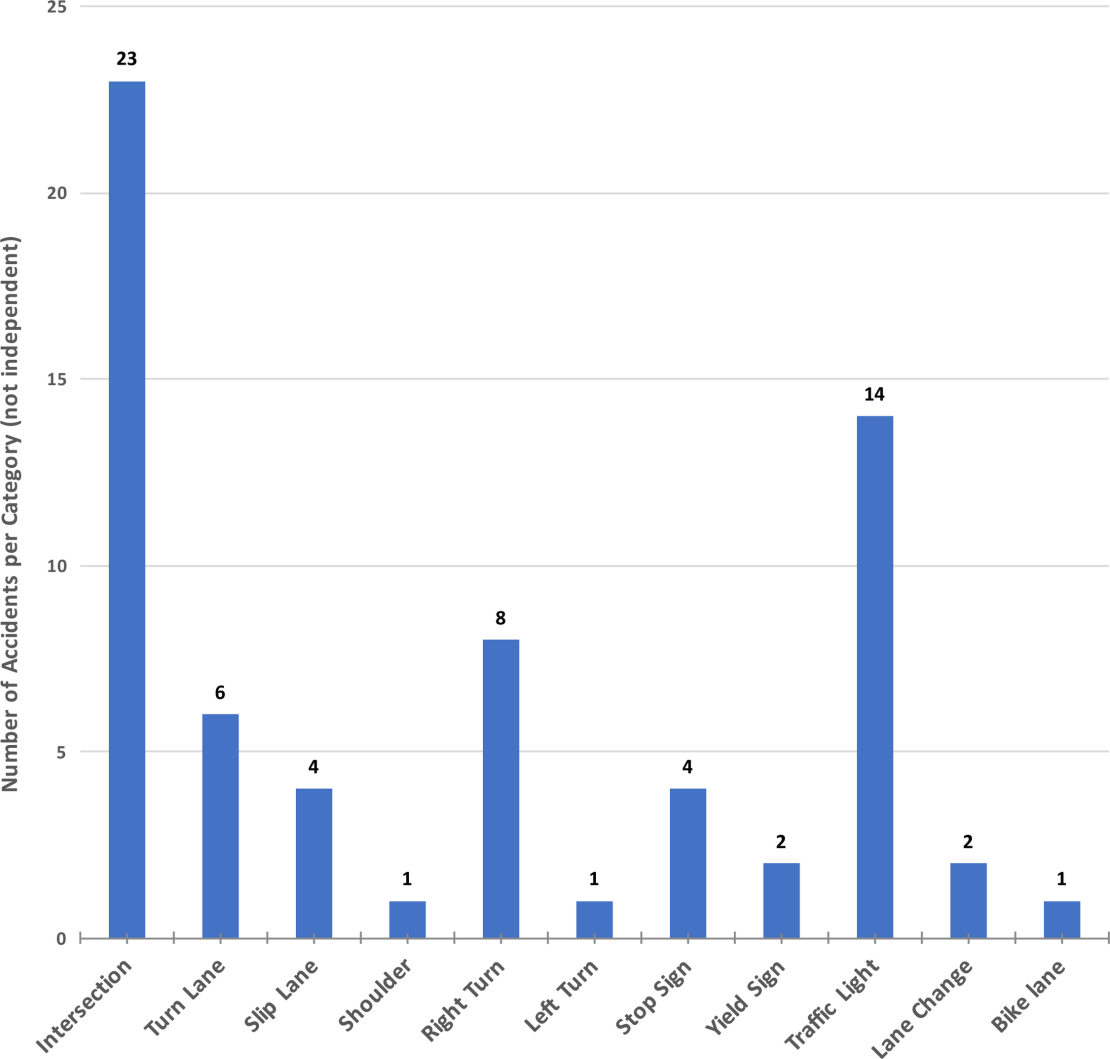
Specific characteristics and locations identified in the AV accident reports. Each column is out of 26 reports; categories are not mutually exclusive.

Finally, an analysis of the location of the accident can be executed. The data contained in [9] shows that 89% of the reported AV accidents happened at an intersection, with a majority of the accidents (48%) occurring in suburban roads, followed by 32% in city roads, and 20% in limited-access roads (highways and expressways). Fig 10 shows additional categories for sites and locations in which the accidents occurred. Note that those categories are not mutually exclusive in general (e.g., right turn or left turn are exclusive, but right turn and shoulder lane are not).

#### 3.2.3 Accidents’ frequency and vehicle make

**Fig 11:**
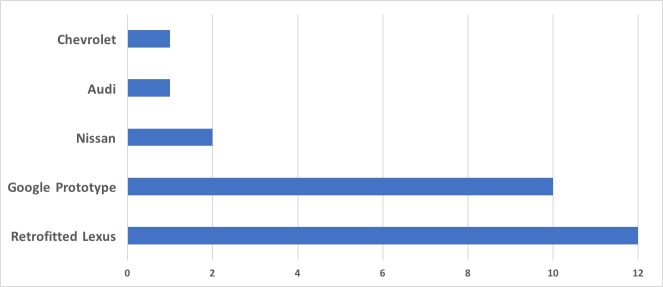
Accidents distribution by AV make.

Fig 11 examines the distribution of the accident reports not by reporter, but by make of the AV involved. Interestingly, the two types of vehicles currently employed by Google have a similar number of accident events.

The Google fleet currently consists of 23 retrofitted vehicles, and 37 prototype design [13]. Fig 11 shows that out of a total of 22 accidents reported by Google, 46% involved Google’s own prototype, and 54% involved the retrofitted Lexus. In 2015, the number of prototypes was up to 50, with 24 vehicles not being driven on public roads [10]. The breakdown of accident frequencies per mileage travelled by vehicle make is an important factor to analyse. Table 2 summarizes the miles travelled by each vehicle make and shows the computed accident frequency per miles travelled and its inverse, i.e. miles driven per accident (average).

**Table 2:** Google's fleet breakdown and accident frequencies.

Type of Vehicle	Total Number of Vehicles	Percentage of Fleet	Percentage of Total Reported Accidents	Total Miles Travelled	Accident Frequency	Miles per Accident
**Google Prototype**	37	61.7%	46%	403,226	2.4e-5	40,322
**Retrofitted Lexus**	23	38.3%	54%	649,841	1.8e-5	54,153

The results of Table 2 indicate that the accident frequency associated to Google’s own prototype are slightly higher than those for the retrofitted Lexus. The reason for such an analysis is to provide evidence to frame in a scientific approach the debate on whether an anthropomorphic design (such as that of Google’s prototype, sporting a rounded shape with a front design that reminds of a human face) might inspire more trust and confidence to drivers of conventional vehicles (as brought forward in [17]), who would thus be less likely to bump accidentally into it. The current data presented in Table 2 does not support such claim, and neither supports the argument that the prototype may be any “safer” (here intended as having a lower accident frequency) than the other make currently tested by Google. Furthermore, Table 3 provides a summary of the accident frequencies computed for the other vehicle makes and accident reporters. The results of Table 3 can serve a similar purpose to those of Table 2, showing that based on current data there is no scientific merit to the idea that conventional vehicles’ drivers might be distracted by the “unusual” shape of the AV or possibly even tempted to test out the AV performance at the expenses of safety with a more aggressive type of behaviour. Google’s vehicles are the most recognizable on the road, but still show a significantly lower accident frequency compared to the other manufacturers. Note however, all estimations of frequency are at this point preliminary, given the small sample size. [18] estimates that a fleet of 100 vehicles would need to be driven accident-free for 12.5 years, 24 hours a day, 365 days a year, to achieve the mileage needed to reliably estimate acceptable fatality rates.

**Table 3:** Accident frequencies by reporters/make of AV accidents.

Type of Vehicle	Total number of Accidents	Total Miles Travelled	Accident Frequency	Miles per Accident
**Nissan (Nissan and GM Cruise)**	2	5,584 + 1,568	2.8e-4	3,576
**Delphi/Audi**	1	19,787	5e-5	19,787
**Chevrolet (GM Cruise)**	1	8,447	1.2e-4	8,447
**Google Prototype**	10	403,226	2.4e-5	40,322
**Retrofitted Lexus**	12	649,841	1.8e-5	54,153

The data presented in this section leads to an average AVs accident frequency of 2.38e-5 (obtained dividing the total number of accidents by the total mileage driven). Based on data from NHTSA and from the Federal Highway Administration (FHWA), it is possible to compute the accident frequency for conventional vehicles in the U.S. for 2015 [19, 20]. The results of the comparison are indicated in Table 4, showing one order of magnitude difference between AVs and conventional vehicles for both accident frequency and its inverse, i.e., the mean mileage driven before accident.

**Table 4:** Comparison of estimated accident frequencies for AV vs. conventional vehicles. Estimate for conventional vehicles is based on [19, 20] which provide updated data until the end of 2015. Data for 2016 and 2017 is still being process by FHWA and NHTSA.

Type of Vehicle	Total number of Accidents	Total Miles Travelled	Accident Frequency	Miles per Accident
**AV**	26	1,088,453	2.38e-5	42,017
**Conventional**	6,296,000	3.148 trillions	2.0e-6	500,000

### 3.3 Accidents’ detection

**Fig 12:**
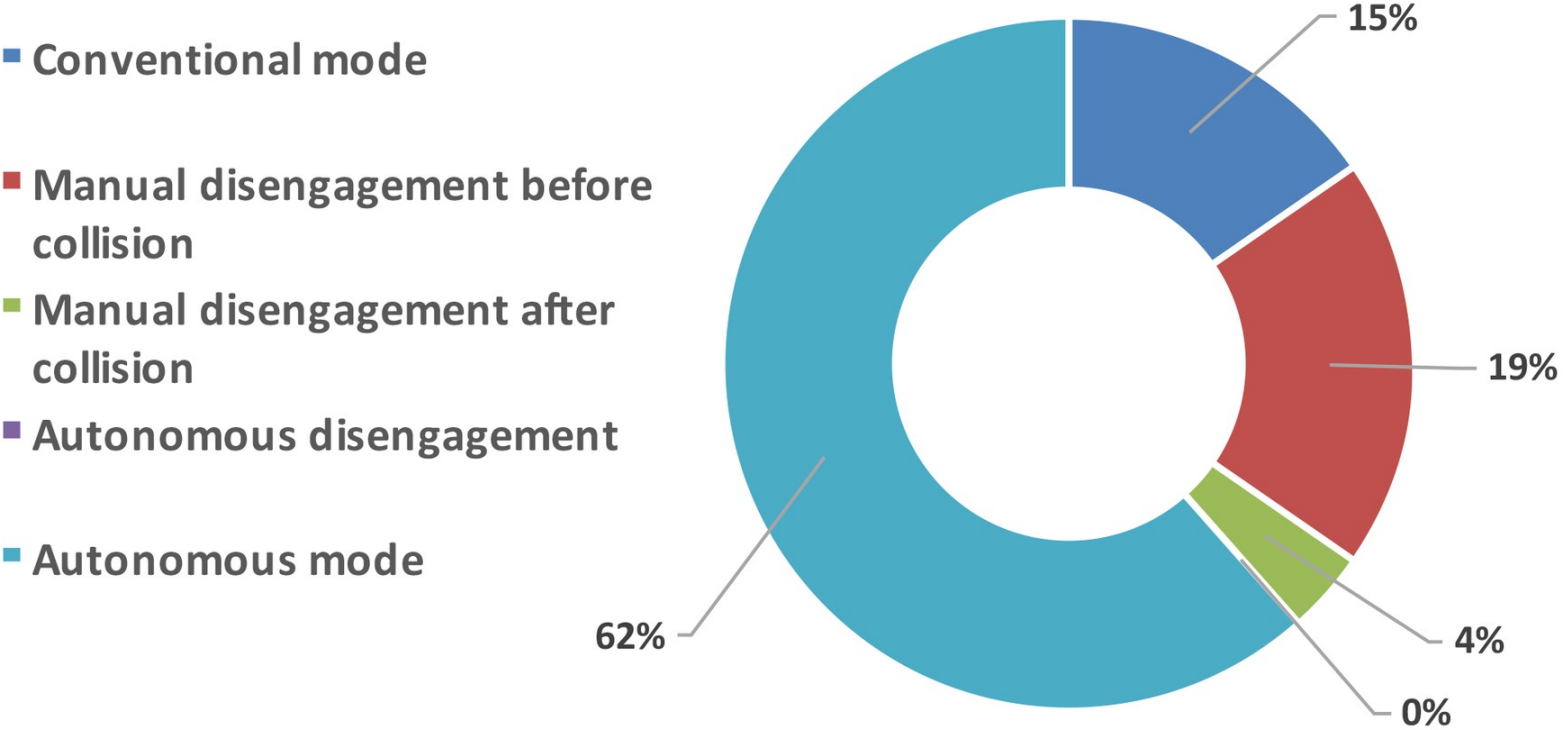
Break-down of accidents in the identified categories.

A careful analysis of the accidents’ descriptions shows that in 22 out of the 26 reported accidents the AV was not-at-fault (a conclusion also highlighted in [12] for the 2014–2015 time span). Additionally, in many instances the AT had been manually disengaged prior to the collision (as indicated in Table 1). In the four situations in which the AV vehicle was at fault, two happened during manual mode (and blame is placed on the human driver in the reports). Fig 12 summarizes the situation that best describes each accident out of the following possible categories (each accident is placed in only one category although they may not look as mutually exclusive):

1. Conventional mode: indicating manual mode was employed before the collision;
2. Manual disengagement before collision: indicating the AT was disengaged by the driver on purpose before the collision occurred;
3. Manual disengagement after collision: indicating the AT was disengaged by the driver on purpose after the collision occurred;
4. Autonomous disengagement: indicating the AT disengaged without intervention from the driver (i.e., actual AT disengagement);
5. Autonomous mode: indicating the AT was not disengaged during the accident sequence.

As can be seen from Fig 12, in no occasion the vehicle underwent an autonomous disengagement (category “e”). This can be indicative of two possible situations: i) a cautious attitude on the part of the trained driver, who attempted to manually disengage the car before the collision (or after the collision, if he/she was not fast enough); ii) the AT was not capable of recognizing and detecting the upcoming collision in time (or at all). As noted in Table 1, and in Figs 6 and 7, 62% of the accidents are “rear-end” fender-bender types. Comparing the accident type with the categories highlighted in Fig 12 leads to the following findings:

1. Rear-end accidents are hard to detect, for both the human driver and the autonomous technology. Whenever the AV driver detected the possibility of a rear-end collision in time, he/she went on to disengage the car. This happened in 38% of the total cases of rear-end collisions.
2. In the 62% remaining rear-end cases, the driver was not able to detect the upcoming collision in time, and neither was the AT, which remained engaged.
3. Out of all the accidents, the AT was capable of detecting and reacting to the upcoming accident only 3 out of 26 times. In all those cases the AT reaction was to attempt breaking, at which point the driver manually disengaged the car and took control.

The last finding can be compared to the conclusions highlighted in [21]. In that study the authors show that drivers have a preference for steering and lane changes input/controls rather than breaking when faced with situations of potential accidents due to acceleration/deceleration mismatches. It is thus interesting to note that in the three situations in which the AT reacted to an off-nominal conditions by attempting to break, the driver took manual control and opted for a different evasive action.

### 3.4 Correlation with mileage driven

**Fig 13:**
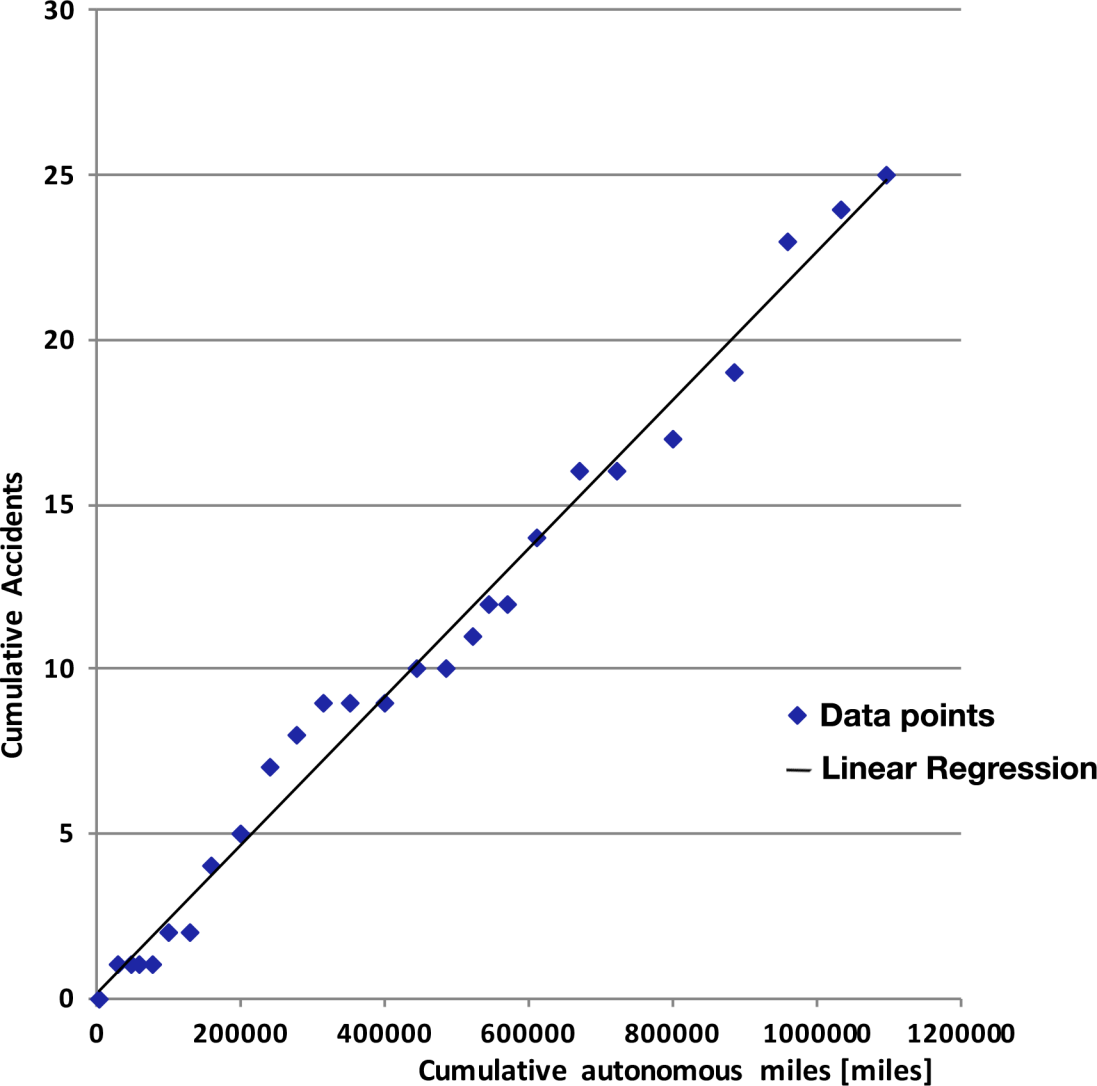
Correlation between cumulative accidents and cumulative autonomous miles. The data shown is only up to December 2016 as some of the manufacturers have yet to provide the cumulative mileage driven for the first part of 2017.

One of the main conclusions drawn in [12] was that the number of accidents observed had a significant high correlation with the autonomous miles traveled (i.e., the more cumulative miles traveled, the more cumulative accidents). This trend remains true for the global analysis that takes into account the accidents from 2014 to 2017. Although the statement may seem evident, it is possible (and desirable) for the cumulative accident trend as a function of cumulative miles to reach a plateau region, signifying that the AV technology is learning from its mistakes and getting close to “accident-free” the more miles traveled. As can be seen in Fig 13, the plateau is far from being reached for now.

The correlation between the cumulative accidents and cumulative autonomous miles is at 0.986 (p-value < 0.001), showing accordance with the results presented in [12]. The hope for a plateau region in the correlation of Fig 13 has deep ties with the technology that powers AVs functioning. Current testing of these vehicles on public roads is used also to the purpose of training the machine learning algorithms that drive the autonomous “brain” of the car. When such algorithms achieve the “fully-tuned” status it will be possible to see that the car is capable of handling more scenarios and avoiding collisions, thus contributing to decreasing the slope of the line shown in Fig 13, and possibly achieving a steady state plateau region with increasing gaps between subsequent accidents when more miles are driven between each adverse event (and thus an increasing mean time between failures).

## 4. Conclusions

The work presented in this paper showed an in-depth analysis of the data contained in accident reports filed to the California Department of Motor Vehicles for accidents involving autonomous vehicles that are undergoing testing on the state’s public roads. The accidents here analyzed were reported between September 2014 and March 2017, and reports were filed by five manufacturers out of the thirty currently holding permits for public testing in California.

The data provided important information on AV accidents dynamics, such as the most recurrent type of accidents, the break-down of damages locations and impact forces, and computed accident frequencies. It was found that rear-end collisions, with the AV standing in front of a conventional vehicle, are the most frequent type of collision, happening with a frequency that doubles that of rear-end “fender-benders” for conventional cars. In 60% of the cases the cars underwent a low impact, with relative speeds below 10 mph. Overall, accident frequencies computed for all manufacturers showed that conventional vehicles drive one order of magnitude more miles compared to AVs before encountering an accident, with a *mean mileage before a crash* for conventional vehicles of about 500,000 miles, compared to 42,017 miles for AVs. Detection and disengagement issues were also analyzed, indicating that the AT technology suffers from the same “deficit” human drivers have in its limitation for detecting and reacting to rear-end type of collisions.

The results presented in this paper are preliminary in nature and leave many fruitful venues for future studies. One of the accomplishments of this research was the creation of a unified database from the fragmented data that is currently publicly available from the California Department of Motor Vehicle. The authors are currently engaged in the definition of safety critical scenarios for testing of human subjects placed in a situation of AT disengagement, with driver-in-the-loop simulation. The analysis presented in this work will inform the creation of such scenarios. Studies of reaction times and responses to disengagements will guide the next steps of the authors’ research.
